# 

**Published:** 1958-10

**Authors:** 


### *Contents*

| | | Page |
|---|---|---|
| THE INVESTIGATION OF PERSISTENT DIARRHOEA | *Dr. John Naish* | 87 |
| MODERN DEVELOPMENTS IN PERIPHERAL VASCULAR SURGERY | *Dr. R. E. Horton* | 92 |
| PERINATAL MORTALITY . . . | *Dr. Thomas E. Oppé* | 99 |
| AN EXHIBITION OF IMPORTANT ANATOMICAL BOOKS | *Mr. A. E. S. Roberts* | 104 |
| HEREDITARY TELANGIECTASIA : HODGKIN'S DISEASE | *Clinical Pathological Conference* | 108 |
| NEWS FROM SOCIETIES . . . . . . | | 113 |
| NOTES AND NEWS . . . . . . . | | 116 |
| APPOINTMENTS . . . . . . . . | | 117 |

